# Heat Tolerance in Curraleiro Pe-Duro, Pantaneiro and Nelore Cattle Using Thermographic Images

**DOI:** 10.3390/ani6020009

**Published:** 2016-01-29

**Authors:** Caio Cesar Cardoso, Flávia Gontijo Lima, Maria Clorinda Soares Fioravanti, Andrea Alves do Egito, Flávia Cristina de Paula e Silva, Candice Bergmann Tanure, Vanessa Peripolli, Concepta McManus

**Affiliations:** 1 Department of Animal Sciences, Faculty of Agronomy and Veterinary Medicine, University of Brasilia (UNB), Darcy Ribeiro Campus, 70910-900 Brasilia, DF, Brazil; cajug29@gmail.com (C.C.C.); flavia_dipaula@yahoo.fr (F.C.P.S.); c.bergmann@gmail.com (C.B.T.); vanessa.peripolli@hotmail.com (V.P.); 2 Veterinary and Animal Science School, Federal University of Goias (UFG), Samambaia Campus, 74690-900 Goiânia, Brazil; flaviamedvet@yahoo.com.br (F.G.L.); clorinda@ufg.br (M.C.S.F.); 3 Embrapa Gado de Corte, Av. Radio Maia, 830, 79106-550, Campo Grande, Brazil; andrea.egito@embrapa.br

**Keywords:** adaptation, adapted breeds, bioclimatology, heat stress, thermography

## Abstract

The objective of this study was to compare physiological and thermographic responses to heat stress in three breeds of cattle. Fifteen animals of each of the Nelore, Pantaneiro and Curraleiro Pe-Duro breeds, of approximately two years of age, were evaluated. Heart and respiratory rates, rectal and surface temperature of animals as well as soil temperature were recorded at 8:30 and 15:30 on six days. Variance, correlation, principal factors and canonical analyses were carried out. There were significant differences in the rectal temperature, heart and respiratory rate between breeds (*p* < 0.001). Nelore and Pantaneiro breeds had the highest rectal temperatures and the lowest respiratory rate (*p* < 0.001). Breed was also significant for surface temperatures (*p* < 0.05) showing that this factor significantly affected the response of the animal to heat tolerance in different ways. The Curraleiro Pe-Duro breed had the lowest surface temperatures independent of the period evaluated, with fewer animals that suffered with the climatic conditions, so this may be considered the best adapted when heat challenged under the experimental conditions. Thermography data showed a good correlation with the physiological indexes, and body area, neck and rump were the main points.

## 1. Introduction

The high temperatures and low humidity during the dry season found in mid-west Brazil directly affect animal production and increased the susceptibility of animal diseases [1,2]. Excessive heat causes decreased food intake and disturbances in protein and energy metabolism, mineral balance, enzymatic reactions, and hormones’ and metabolites’ secretion in the blood [3,4]. All these factors negatively affect production, causing economic losses for the farmer. One alternative is the use of animals adapted or tolerant to the local conditions in the producing regions [5,6]. Adapted animals are characterized by the ability to survive in extreme environmental and climatic conditions. These animals maintain or show a minimal reduction in productive performance exhibiting high reproductive efficiency, disease resistance, longevity and low mortality rate during the exposure to stress [7].

The Pantaneiro breed is a descendant of European cattle and played an important role in the economy of the Pantanal flooded regions until the early twentieth century [8]. Adapted to the nutritional status of flooded lowland, food shortages and through a long process of natural selection and adaptation, this rusticity has allowed the survival of this breed [9] showing advantage over the zebu cattle in the same region [10]. The Curraleiro Pe-Duro cattle is typical of the Brazilian Cerrado (Savannah). This breed was reared under diverse farming conditions, with minimum health care and nutrition, resulting in animals resistant to endoparasites and ectoparasites, and low production costs [11]. In common, these two breeds show rusticity, regional disease resistance, ability to tolerate long periods of dietary restriction and rapid recovery of body weight after periods of feed restriction [12]. Due to natural selection in adverse conditions, especially nutritional aspects, these animals are generally smaller compared to commercial breeds [13,14] and can take up to four years to be slaughtered in diverse farming systems [11].

In the early twentieth century, Zebu (*Bos indicus*) cattle were imported into Brazil in an attempt to increase the productivity of locally adapted Brazilian cattle which were mainly *Bos taurus* [13]. At present, most of the Brazilian locally adapted breeds have been substituted by breeds of zebu origin, mainly Nelore.

Adaptability evaluation and heat tolerance of the animals are mainly determined by the physiological parameters such as respiratory rate and body temperature [15]. Restraint and handling procedures are required for the measurement of this parameter that can cause a stress response changing the results [16]. New tools, such as infrared thermography, are alternatives to assess the impact of environmental factors on thermal stress in animals. Thermographic images may indicate circulatory changes induced by increased body temperature related to environmental heat stress by surface temperatures of the animals. The main benefits of this tool are improving animal welfare during the evaluation and gaining a larger number of evaluations in a shorter time, and without animal restraint, it has been used as an indicator of rectal and vaginal temperatures [17]. 

The objective of this study was to compare physiological and thermographic responses to heat stress in locally adapted (Pantaneiro and Curralero Pe-duro) and exotic (Nelore) cattle breeds.

## 2. Experimental Section

Animal care procedures throughout the study followed protocols approved by the Ethics Committee for Animal Use (CEUA) at the University of Brasilia, number 33/2009.

The experiment was carried out over 97 days on an experimental farm belonging to the Federal University of Goias in São Francisco de Goias city, in the Brazilian mid-west Cerrado, located at 15°55′50″ South and 49°15′40″ West. The climate is classified as a tropical climate with a defined dry season. Forty-five males approximately two years old, 15 per breed (Pantaneiro, Curraleiro Pé-duro and Nelore) were used. The animals were reared as one group with the same environmental and treatment conditions and were fed with a 70% roughage and 30% concentrate diet on a dry matter basis. For the duration of the experiment, animals were maintained in a roofless corral with lateral wooden slating.

Two hours before physiological collections, the animals were taken to an open corral without shade and contained in a chute. Animal surface and soil temperatures were measured by infrared thermograph ThermaCAM^®^ model T400 (FLIR Systems Inc., Wilsonville, OR, USA). This camera has infrared resolution 320 × 240 pixels, with thermal sensitivity of <0.05 °C at 30 °C (86 °F)/50 mK. Two images were taken from each animal at a distance of 1.5 m, one laterally of the whole body and the other of the head region. These were taken calmly to a covered area immediately after physiological measurements, so no time was allowed for the animals to adapt to the shaded area before recordings were completed. This procedure was adopted because sunlight on the animals alters the conductivity and emissivity of the thermal image [18]. 

The physiological parameters measured included: respiratory rate, heart rate and rectal temperature. Heart rate was measured using a stethoscope, the respiratory frequency was obtained through observation of respiratory movements and the rectal temperature was measured with a digital thermometer introduced into the animal’s rectum. Heart and respiratory rates, rectal and body and head region surface temperatures of animals as well as the soil temperature were recorded at 08:30 and 15:30 h. The procedure was repeated on six separate days, over a three-week period at the end of the experiment. 

Standard Quickreport^®^ tools were used for analysis of the images: tool “line” was used to obtain the average temperature of the muzzle, head and neck regions of the animals. The tool “point” was used to obtain the highest temperature in the axilla, groin and rump of the animals, as well as the tool “area” was used to measure the average temperature of the whole body, the muzzle and chamfer regions, and the temperature of two distinct areas of the soil near the animals.

The black globe temperature in the sun (BGsun-°C) and the shade (BGShade-°C) were taken using a mobile globe thermometer ITWTG-2000 (INSTRUTEMP, Measuring Instruments Ltda, SP, BR) at the experimental site. The environment temperature (°C), relative humidity (%) and wind speed (m/s) were obtained from a local weather station ITWH1080 (INSTRUTEMP, Measuring Instruments Ltda., SP, BR) at the experimental site.

Temperature and Humidity Index (THI) was calculated according to the following National Research Council formula [19]: THI = (1.8 × Tdb + 32) − (0.55 − 0.0055 × RH) × (1.8 × Tdb − 26), where, Tdb: dry bulb temperature (°C) and RH: relative humidity (%).

Statistical analyzes were performed using the Statistical Analysis System^®^ package (v.9.3, SAS Inc., Cary, NC, USA). Analysis of variance (PROC GLM) was carried out to evaluate the effect of the soil, environment and black globe temperatures as well as THI, air humidity, wind speed, period of the day and the genetic group of the animals on the body temperatures and physiological measures. The associations between traits were investigated using correlation analysis (CORR) and principal component analysis to (PRINCOMP) attempt to understand the sources of variation in the data. The between-class variation was investigated using a canonical analysis (CANDISC).

## 3. Results

In the present study, the lowest temperature recorded was 20 °C in the morning and the highest temperature was 36.5 °C in the afternoon. Relative humidity ranged between 22% and 77% and the wind speed was 0.6 m/s. The black globe temperatures in the shade and in the sun ranged between 20.5 °C–36.5 °C and 20.5 °C–53.5 °C, respectively, and the temperature and humidity index average was 76.23 (mild stress). The soil temperature average was 31.57 °C.

Breed affected all physiological parameters and all surface temperatures (Table 1). The Curraleiro Pe-duro breed had the lowest body area, neck, rump, head, groin and axilla temperatures as well as rectal temperature and the highest respiratory rate. Nelore breed had the lowest heart rate.

**Table 1:** Surface temperatures and physiological parameters of the breeds.

Breed	Body Area	Neck	Rump	Muzzle	Head	Groin	Axilla	HR	RR	RT
Pan	36.67 ^a^	36.49 ^a^	36.50 ^a^	34.19	36.50 ^a^	36.16 ^a^	36.07 ^a^	117.52 ^a^	43.33 ^b^	39.05 ^b^
Nel	36.45 ^a^	36.41 ^a^	36.83 ^a^	34.31	36.35 ^a^	36.33 ^a^	36.43 ^a^	103.91 ^b^	42.89 ^b^	39.43 ^a^
Curr	35.34 ^b^	35.36 ^b^	35.14 ^b^	33.5	35.38 ^b^	34.79 ^b^	35.14 ^b^	117.37 ^a^	46.55 ^a^	38.63 ^c^
SEM	0.15	0.15	0.19	0.33	0.22	0.16	0.16	1.15	0.56	0.03
Pr > F	***	*	**	*	**	***	*	***	***	***
RV								36.0–60.0	26.0–50.0	36.7–39.1

Pan: Pantaneiro; Nel: Nelore; Curr: Curraleiro Pe-Duro; RV: reference value [20]; HR: heart rate; RR: respiratory rate; RT: rectal temperature; SEM: standard error of the mean. Means with different letters in the column differ at 5% by Tukey test. *: *p* < 0.05; **: *p* < 0.01; ***: *p* < 0.001.

The period of the day influenced the surface temperatures, as well as respiratory and heart rate of the animals (Table 2) as expected due to natural variation in temperature throughout the day, which was on average 16.5 °C per day. In the morning period, the Curraleiro Pe-Duro breed showed the lowest values of body area, neck, rump, muzzle, head, groin and axilla temperatures, compared to the other breeds. In the afternoon period, little difference in the surface temperatures was observed among the breeds studied. For the physiological parameters, the Nelore breed showed the lowest heart rate compared with the other breeds, both in the morning and in the afternoon, as well as the higher rectal temperature and the lowest respiration rate, in the afternoon period. For the Curraleiro Pe-Duro breed, the respiratory rate was higher compared to the other breeds, especially in the afternoon. This breed had the lowest rectal temperature.

The average temperatures in the afternoon (Table 2) were higher than in the morning for all points evaluated as expected due to the greater environmental challenge for the animals, such as higher ambient temperature in the afternoon.

**Table 2:** Surface temperatures and physiological parameters of the breeds in the morning and afternoon.

Breeds	Body Area	Neck	Rump	Muzzle	Head	Groin	Axilla	HR	RR	RT
	Morning									
Pan	34.50 ^a^	34.91 ^a^	33.94 ^b^	30.95 ^a^	33.64 ^a^	34.13 ^a^	33.11 ^b^	115.20 ^a^	39.20 ^b^	38.82 ^b^
Nel	34.89 ^a^	34.91 ^a^	35.19 ^a^	32.22 ^a^	34.30 ^a^	34.69 ^a^	34.93 ^a^	103.82 ^b^	42.04 ^a^	39.37 ^a^
Curr	32.87 ^b^	33.22 ^b^	31.77 ^c^	29.07 ^b^	31.64 ^b^	32.22 ^b^	32.74 ^c^	118.48 ^a^	42.75 ^a^	38.30 ^c^
SEM	0.15	0.14	0.21	0.33	0.23	0.17	0.16	1.69	0.66	0.05
Pr > F	***	***	***	***	***	***	***	***	**	***
	**Afternoon**									
Pan	38.84 ^a^	38.50 ^a^	39.05	37.65	39.57 ^a^	38.25 ^a^	38.35 ^a^	119.55 ^a^	47.46 ^ab^	39.29 ^b^
Nel	38.02 ^b^	37.91 ^ab^	38.47	36.47	38.49 ^b^	37.98 ^ab^	37.93 ^ab^	104.00 ^b^	43.73 ^b^	39.48 ^a^
Curr	37.81 ^b^	37.50 ^b^	38.52	37.67	38.71 ^b^	37.36 ^b^	37.54 ^b^	116.55 ^a^	50.35 ^a^	38.96 ^c^
SEM	0.11	0.14	0.13	0.40	0.14	0.11	0.13	1.85	0.84	0.03
Pr > F	**	**	NS	NS	**	**	*	***	**	***
**Time**	**Mean**									
Morning	34.08 ^b^	34.20 ^b^	33.64 ^b^	30.79 ^b^	33.25 ^b^	33.68 ^b^	33.82 ^b^	112.50	41.33 ^b^	38.83 ^b^
Afternoon	38.22 ^a^	37.97 ^a^	38.68 ^a^	37.25 ^a^	38.91 ^a^	37.86 ^a^	37.94 ^a^	113.17	47.18 ^a^	39.24 ^a^
SEM	0.11	0.12	0.14	0.34	0.16	0.12	0.12	1.18	0.66	0.03
Pr > F	***	***	***	***	***	***	***	NS	***	***

Pan: Pantaneiro; Nel: Nelore; Curr: Curraleiro Pé-Duro; HR: heart rate; RR: respiratory rate; RT: rectal temperature; SEM: standard error of the mean. Means with different letters in the column differ at 5% by Tukey test. NS: not significant; *: *p* < 0.05; **: *p* < 0.01; ***: *p* < 0.001.

The correlation between the surface and the environment temperatures was high and positive (Table 3). In general, respiratory and heart rates showed low and positive correlations with other traits. Rectal temperature showed a low correlation with respiratory and heart rates. THI had a high and positive correlation with air temperature. Humidity had a negative correlation with surface and air temperatures (−0.97).

**Table 3:** Correlations between superficial temperatures and physiological traits of the breeds.

	Soil	Air	RH	WS	BGshade	BGSun	THI	Body Area	Neck	Muzzle	Head	Groin	Rump	Axilla	HR	RR
Air	0.78 ***															
RH	−0.73 ***	−0.97 ***														
WS	0.75 ***	0.61 ***	−0.58 ***													
BGShade	0.82 ***	0.99 ***	−0.97 ***	0.66 ***												
BGSun	0.51 ***	0.79 ***	−0.82 ***	0.41 ***	0.78 ***											
THI	0.79 ***	0.97 ***	−0.90 ***	0.60 ***	0.96 ***	0.74 ***										
Body Area	0.84 ***	0.86 ***	−0.83 ***	0.60 ***	0.88 ***	0.66 ***	0.84 ***									
Neck	0.78 ***	0.82 ***	−0.79 ***	0.57 ***	0.83 ***	0.64 ***	0.79 ***	0.93 ***								
Muzzle	0.65 ***	0.67 ***	−0.64 ***	0.49 ***	0.68 ***	0.50 ***	0.65 ***	0.69 ***	0.63 ***							
Head	0.82 ***	0.84 ***	−0.82 ***	0.59 ***	0.86 ***	0.64 ***	0.82 ***	0.93 ***	0.88 ***	0.68 ***						
Groin	0.77 ***	0.87 ***	−0.85 ***	0.53 ***	0.88 ***	0.68 ***	0.84 ***	0.92 ***	0.87 ***	0.64 ***	0.87 ***					
Rump	0.85 ***	0.86 ***	−0.83 ***	0.60 ***	0.88 ***	0.68 ***	0.85 ***	0.95 ***	0.87 ***	0.68 ***	0.91 ***	0.89 ***				
Axilla	0.81 ***	0.83 ***	−0.80 ***	0.57 ***	0.85 ***	0.64 ***	0.81 ***	0.93 ***	0.88 ***	0.64 ***	0.87 ***	0.89 ***	0.89 ***			
HR	0.14 **	−0.004 ^NS^	0.04 ^NS^	0.11 ^NS^	0.01 ^NS^	−0.21 **	0.03 ^NS^	0.08 ^NS^	0.04 ^NS^	−0.02 ^NS^	0.07 ^NS^	0.06 ^NS^	0.06 ^NS^	0.07 ^NS^		
RR	0.31 ***	0.31 ***	−0.29 ***	0.26 ***	0.32 ***	0.21 **	0.30 ***	0.40 ***	0.36 ***	0.32 ***	0.40 ***	0.33 ***	0.37 ***	0.38 ***	0.15 **	
RT	0.47 ***	0.57 ***	−0.53 ***	0.21 **	0.57 ***	0.56 ***	0.61 ***	0.58 ***	0.57 ***	0.33 ***	0.53 ***	0.59 ***	0.59 ***	0.53 ***	0.05 ^NS^	0.21 **

Air: air temperature; WS: wind speed; RH: relative humidity; Soil: soil temperature; BG: black globe temperature; THI: temperature and humidity index; HR: heart rate; RR: respiratory rate; RT: rectal temperature. ^NS^: not significant; *: *p* < 0.05; **: *p* < 0.01; ***: *p* < 0.001.

**Figure 1:**
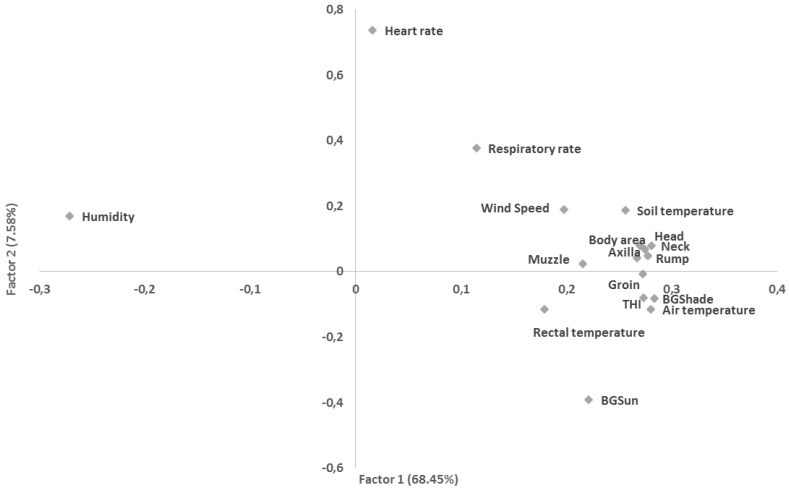
Principal factor analysis for surface temperatures, physiological and environmental parameters analyzed. BGSun: black globe temperature in the sun; BGSha: black globe temperature in the shadow; body area, head, neck, axilla, rump, muzzle and groin surface temperatures; THI: temperature and humidity index.

Figure 1 shows the principal factor analysis for surface temperatures, and physiological and environmental parameters analyzed. In the first autovector it was observed that the surface temperatures were related to the environmental temperature. The only exception was the muzzle temperature. In the second autovector, it was observed that when the ambient temperature increased, rectal and black globe temperatures also increased. Increased ambient temperature was followed by increased heart and respiratory rates. Increase in humidity was associated with a decrease in air temperature.

**Figure 2:**
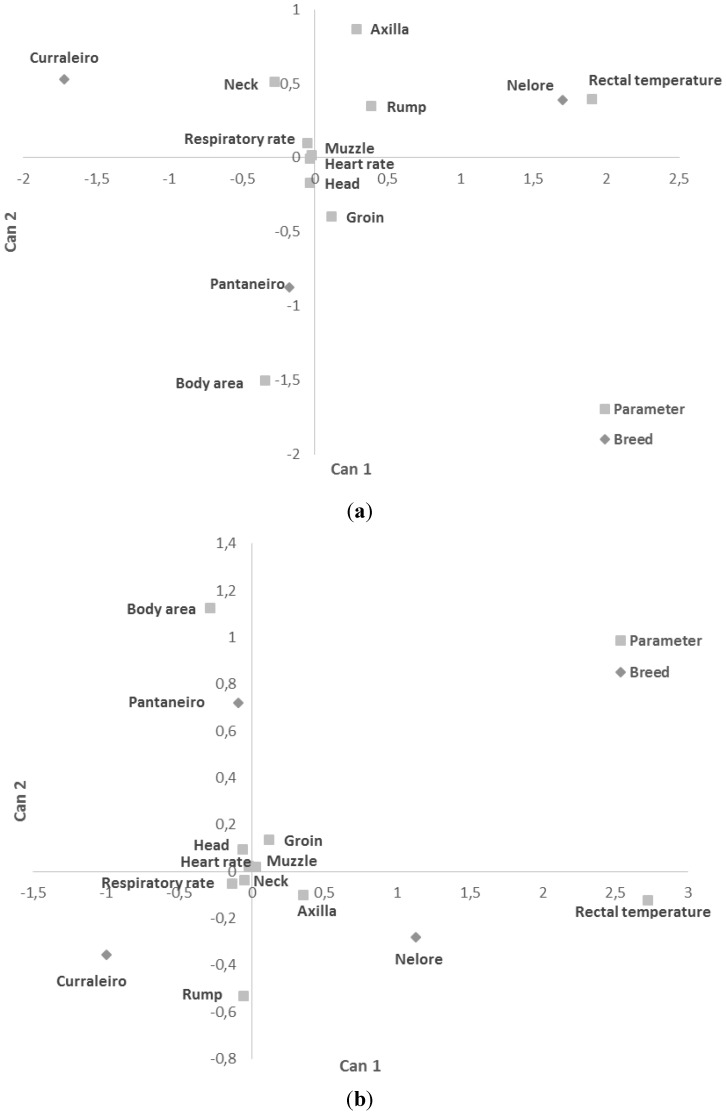
Mean canonical discriminant analysis by breed with discriminating parameters in the morning (**a**) and in the afternoon (**b**). Body area, head, neck, axilla, rump, muzzle and groin surface temperatures.

Nelore breed presented higher rectal temperature and axilla temperatures both in the morning and in the afternoon (Figure 2), while Pantaneiro had higher body area temperature and Curraleiro Peé-duro higher respiratory rate.

The physiological response differences among breeds can also be seen by the percentage of samples that were above the reference values for rectal temperature and respiratory and heart rates (Table 4). In the morning, all Curraleiro Pe-Duro had rectal temperatures below the reference value (36.7 °C–39.1 °C, [20]). The heart rate of all Pantaneiro breed presented values above the reference values (36.0–60.0 mov/min, [20]) both in the morning and afternoon. In all three breeds, more than 90% of the collections of respiratory rates were above the reference values (26–50 breaths/min, [20]). In the afternoon, Curraleiro Pe-Duro breed had a low proportion of animals with rectal temperatures above the reference values, while the Pantaneiro breed had 18%. The Nelore breed had a higher percentage of animals above the reference values for rectal temperature. 

**Table 4:** Percentage of animals within each genetic group above reference values for respiratory rate (RR) heart rate (HR) and rectal temperature (RT).

Group	RR	HR	RT
	**Morning**		
Curraleiro	78	82	0
Nelore	89	87	29
Pantaneiro	100	76	7
		**Afternoon**	
Curraleiro	96	98	2
Nelore	98	91	40
Pantaneiro	100	98	18

## 4. Discussion

Animals can suffer heat stress when the temperature is higher or lower than the thermal comfort zone [21], and also according to Nienabar & Hahn [22], night-time cooling is fundamental for an animal to maintain production under heat stress. In this experiment, temperatures above the thermal comfort zone of cattle (27 °C) were recorded every day during the experiment. 

Baccari Junior in 1990 [7] stated that the adaptability of animals to hot environments can be measured by testing the physiological adaptability and tolerance to heat. The criteria of tolerance and adaptation of animals are determined mainly by respiratory rate and body temperature [23]. The biggest problem with these methods using physiological indicators of stress is that containment is necessary and handling procedures can cause a stress response, thereby altering the results [24]. Thus, the use of infrared thermography can assist in identifying the thermal stress of the animals. 

Body area temperature using thermography provides a larger area for analysis, giving mean values for the whole body, but also provides a larger area that can be affected by external factors such as soil and air temperatures. Thus, there is a greater temperature gradient than at specific points. The environmental variables such as solar radiation incidence and wind speed can negatively influence the body area temperature, thus requiring more care [25]. The Curraleiro Pe-Duro animals have a larger body surface per unit of body weight resulting in higher heat exchange with the environment [26]. Fitzhugh [27] stated that the body size may have important biological advantages regarding aspects related to adaptation. This would explain the lower body area temperatures found in the Curraleiro Pe-duro breed when compared with the Nelore and Pantaneiro breeds. Also, according to Bianchini *et al.* (2006) [14], the Curraleiro Pe-Duro breed had a higher percentage of tissue area of the secretory glands and transpiration rate compared to the Nelore breed. Differences between surface temperatures can be justified by lower pigmentation of the Nelore coat which tends to be white or grey in comparison with the other two breeds which have a reddish coat. This coat pigmentation is an important protective factor against ultraviolet rays [28], but previous studies on heat tolerance with these breeds [29] showed that coat and skin traits explained little variation in physiological parameters.

Curraleiro Pe-duro animals had the lowest rectal temperature and was the only breed that remained within the reference values for cattle (36.7 °C–39.1 °C, [20]), but showed the highest respiratory rate. This may be due to water evaporation which absorbed heat and released it through respiration. The animal increased its breathing rate, trying to increase the loss of excessive heat and maintain a low rectal temperature [30]. The Pantaneiro had the highest heart rate as these animals show increased cutaneous circulation, which in turn facilitates heat loss by convection and radiation due to cutaneous vasodilation [31]. To compensate the increase in blood flow to the skin, the heart rate increased [2]. 

Heart rate was similar between the periods of day and the values were above the normal physiological parameters [20]. One reason may be the physical restraint stress for data collection, once when the animal feels cornered, there is interference in the security comfort zone [16]. Thus, the animal becomes stressed, which can lead to the release of stress-related hormones such as adrenaline and cortisol raising the blood pressure and the number of heartbeats [32]. These hormones have a beneficial effect on keeping the animal alert to escape, but cause tachycardia by direct action on the cardiac muscle causing spleen contraction providing more red cells in the circulation, and causing increased vascular pressure and other deleterious effects in the animal such as reduced immunity [33].

The correlation between the surface and the environment temperatures was high and positive and agreed with the results obtained by Knizkova *et al.* [34]. George *et al.* [17] also found low correlations with thermographic muzzle and rectal temperatures. Rectal temperature showed a low correlation with respiratory and heart rates, and was not in agreement with results obtained by McManus *et al.* [6], who found a high correlation (0.70) between these factors. When the environmental temperature increases, the surface and black globe temperatures also increased. The higher the air humidity, the less evaporative water loss, making animal cooling slower. The lower water concentration in the air provides quicker animal cooling, due to the increased water evaporation rate through the skin and the respiratory system [28,35]. The region where the experiment was carried out has high daily temperatures but very low humidity; therefore, unlike the humid tropics, moisture evaporation from the animal body surface is still possible.

In this study, it was observed that the surface temperatures were related to the environmental temperature, except for the muzzle temperature. According to Paim *et al.* [20], this is because the muzzle surface temperature reflects the temperature of the expired air. Considering the use of infrared temperatures, those recorded at rump, neck and flank regions were the most effective in determining the body temperatures.

The higher rectal and axilla temperatures both in the morning and in the afternoon in the Nelore cattle, the higher body area temperature in the Pantaneiro and the higher respiratory rate in Curraleiro may indicate different means of liberating heat in these animals as they developed in different regions of the country under different environmental conditions [6]. The differences between morning and afternoon measurements observed in this study were generally greater in the locally adapted breeds compared to Nelore breed.

## 5. Conclusions

The locally adapted breeds in general, had the lowest rectal temperatures, but had higher respiratory and heart rates than Nelore. The Curraleiro Pe-Duro breed had the lowest rectal and surface temperatures being the best adapted when heat challenged. Thermography data showed a good correlation with the physiological indexes, and body area, neck and rump were the main points.
